# Looking back to inform the future: a review of published paramedicine research

**DOI:** 10.1186/s12913-022-08893-4

**Published:** 2023-02-02

**Authors:** N. Cavanagh, I. E. Blanchard, D. Weiss, W. Tavares

**Affiliations:** 1 Alberta Health Services, Emergency Medical Services, Edmonton, Alberta Canada; 2 Department of Community Health Sciences, University of Calgary, Cumming School of Medicine, Calgary, Alberta Canada; 3 The Wilson Centre, Department of Medicine, University of Toronto/University Health Network, Toronto, Ontario Canada; 4 Department of Health and Society, University of Toronto, Toronto, Ontario Canada; 5 York Region Paramedic and Senior Services, Community Health Services Department, Regional Municipality of York, Newmarket, Ontario Canada

**Keywords:** Paramedicine, Mixed methods review, Emergency medical services, Ambulance, Professional practice

## Abstract

**Objective:**

Paramedicine has evolved in ways that may outpace the science informing these changes. Examining the scholarly pursuits of paramedicine may provide insights into the historical academic focus, which may inform future endeavors and evolution of paramedicine. The objective of this study was to explore the existing discourse in paramedicine research to reflect on the academic pursuits of this community.

**Methods:**

We searched Medline, Embase, CINAHL, Google Scholar and Web of Science from January, 2006 to April, 2019. We further refined the yield using a ranking formula that prioritized journals most relevant to paramedicine, then sampled randomly in two-year clusters for full text review. We extracted literature type, study topic and context, then used elements of qualitative content, thematic, and discourse analysis to further describe the sample.

**Results:**

The initial search yielded 99,124 citations, leaving 54,638 after removing duplicates and 7084 relevant articles from nine journals after ranking. Subsequently, 2058 articles were included for topic categorization, and 241 papers were included for full text analysis after random sampling. Overall, this literature reveals: 1) a relatively narrow topic focus, given the majority of research has concentrated on general operational activities and specific clinical conditions and interventions (e.g., resuscitation, airway management, etc.); 2) a limited methodological (and possibly philosophical) focus, given that most were observational studies (e.g., cohort, case control, and case series) or editorial/commentary; 3) a variety of observed trajectories of academic attention, indicating where the evolution of paramedicine is evident, areas where scope of practice is uncertain, and areas that aim to improve skills historically considered core to paramedic clinical practice.

**Conclusions:**

Included articles suggest a relatively narrow topic focus, a limited methodological focus, and observed trajectories of academic attention indicating where research pursuits and priorities are shifting. We have highlighted that the academic focus may require an alignment with aspirational and direction setting documents aimed at developing paramedicine. This review may be a snapshot of scholarly activity that reflects a young medically directed profession and systems focusing on a few high acuity conditions, with aspirations of professional autonomy contributing to the health and social well-being of communities.

## Background

Paramedicine is a rapidly evolving profession that encompasses not only paramedics working in emergency and non-emergency roles [1, 2], but for the purposes of this study the system in which paramedics work.^1^ There is a question globally as to whether the changes within paramedicine may now outpace the evidence that informs it [3–5], which is especially evident in Canada.

National efforts to guide change in paramedicine have in some cases been in the form of reports proffering new directions but limited in the evidence used to support these recommendations. For instance, in Canada the 2006 Emergency Medical Services (EMS) Chiefs of Canada, now Paramedic Chiefs of Canada (PCC), disseminated a report entitled “The Future of EMS in Canada: Defining the New Road Ahead” [6]. Community paramedicine, or the provision of community care by specially trained paramedics, was recommended in the report. However, Bigham et al. in a systematic review published 7 years after the report included only 11 community paramedicine publications [7], and Thurman et al. [8] in a scoping review 14 years later included 29 publications. Both studies argued that making conclusions about the value and effectiveness of community paramedicine programs is difficult given the paucity and rigor of available evidence. Since 2006, research capacity and productivity in paramedicine has expanded [9], and evidence-based approaches are more common and expected. As a result, today it is more likely that any existing and future directions are guided by evidence, but in what areas and to what extent research or evidence supports or aligns with emerging directions for the profession remains unclear.

In Canada, at least two seminal documents related to supporting knowledge production and future directions for the profession have been published since 2006. One is a report titled the “Canadian National EMS Research Agenda” (2013) [10]. This research agenda was based on a mixed methods study [4, 11–13] and came about from the recognition of the need for evidence to drive decision-making related to clinical care and system-wide policy decisions. It provided a targeted effort to build the research enterprise in paramedicine, and made 19 recommendations in five categories (time, opportunities, and funding; education and mentorship; culture of research and research collaboration; structure, process, and outcomes; and future directions). It also achieved consensus from experts on 36 topics that required increased research effort.

The second is a recent publication titled “Principles Guiding the Future of Paramedicine in Canada” (2021) [14]. This publication was based on a qualitative study and recognized the broad changes that were occurring nationally in models of care, scope of practice, and policy. It also recognized that the 2006 visioning document was now outdated, and that the system was in need of a consolidated and shared framework to effectively guide future directions for the profession. It identified 10 principles:

- healthy professionals,
- professional autonomy,
- integrated healthcare framework,
- social responsiveness,
- continuous learning environment,
- quality based framework,
- patients and communities first,
- evidence informed practice and systems,
- intelligent distribution of services,
- and healthcare along a health and social continuum.

These principles promote more accountability to the professions of paramedicine, and the public and the healthcare system it intends to contribute to.

Collectively these publications, along with other published material such as standards developed by the Canadian Standards Association [15], have influenced, and are influencing the trajectory of paramedicine in Canada and other countries. However, as paramedicine strives to align its activities with the strongest empirical evidence, tracking the narrative on what is being published, and understanding where the paramedicine community is positioning its academic capital is needed. Recent bibliometric studies have highlighted where citations are greatest, and what journals, geographical location, methodologies, and contributors they represent, but they have yet to explicitly focus on the conversations and content of the citations [9, 16]. Collectively, these types of literature summaries can provide additional insights into the academic priority of the paramedicine research community, the state of the evidence base informing its advances, and where there may be gaps supporting its intended and unintended growth or evolution. Our aim is to contribute to this growing reflection on paramedicine’s academic pursuits.

The objective of this literature review was to broadly support initiatives to create a new vision for paramedicine in Canada by exploring the existing discourse in paramedicine research to reflect on the academic pursuits of this community. The research question guiding our work was: What research pursuits are being engaged in by the paramedicine community? Attending to the academic discourse provides a means of reflecting on whether existing or new directions can be supported by an evidence base and where additional attention may be necessary.

## Methods

Our goal was to conduct a review of paramedicine research through use of a wide-ranging search strategy and elements of discourse analysis to contribute to a discussion of the most prevalent topics. As this review attempted to explore emergent discourse across an expansive literature with varying publication sources, we required a methodology that would: 1) provide a degree of rigor in search and selection of this broad field of potential literature; and 2) allow flexibility and iteration in the process around determining which literature was most relevant to our research objective. To structure our search, selection, and analysis we turned to the 2016 discourse analysis by Rangel et al. This research team had the similar task of considering an expansive literature (50 years’ worth) in medical education [17].

Rangel et al., applied a discourse analysis to “identify emergent thematic trends, the use of words and concepts, and how they are made and used by persons and institutions…”. Our aim was to examine the discourse derived from the academic pursuits of the paramedicine community, while using a systematic approach. When “a discourse is prominent it will be replicated and reproduced, and so it will be possible to pick up and trace its origins and evolution through a period of time” [17]. In this study, we identified articles focusing on paramedicine, then examined a sample of these articles using elements of discourse analysis as outlined by JP Gee [18] and leveraged by Rangel et al. [19]. To further guide our analysis we also used qualitative content and thematic analysis to help group and categorize topic areas [20–22].

### Search strategy

In collaboration with a research librarian, we searched English language journals using Medline and Embase (Ovid interface), and CINAHL (EBSCO interface) from January, 2006 to April, 2019. We used a start date for the search of 2006 to align with the publication of the initial PCC report [23]. As the search was intended to identify the breadth of subjects in the literature related to paramedicine rather than a specific topic, broad search terms were used. These terms were based on common search terms employed in paramedicine [24, 25], and included “prehospital”, “paramedicine”, “emergency medical services”, “paramedic”, and “ambulance”; terms were combined using the Boolean operator “OR”. We also conducted a reverse citation search that identified literature citing the 2006 PCC report using Google, Google Scholar, and Web of Science.

### Selection of articles

We anticipated that the total citation yield would be excessively large, and it would be neither feasible nor necessary to review all of it. We determined that a “systematic cluster sample” approach like that taken by Rangel et al. would be appropriate as it would identify articles relevant to our topic, but not assume that topics occur uniformly over time; an assumption that could end up excluding or underrepresenting topics that become prevalent for a particular and finite period.

Our first step was to reduce the yield by identifying the citations that were most relevant to paramedicine through application of the above-mentioned search terms to titles and abstracts. Then, we took the reduced yield and stratified by journal, identifying the journals that most frequently published paramedicine relevant citations. Finally, we took a sample of articles from those journals by grouping all citations into two-year clusters from 2006 to 2017, and each of 2018 and 2019, and taking a sample from each journal in each cluster. To include as much potential discourse as possible, we also elected to include all types of review articles. Based on the results of the cluster sampling, we further elected to include the top six peer reviewed journals. “Top” journals were identified by how many relevant citations were retrieved relative to the journal’s total citations, the impact factor, and the country of publication. In addition to peer reviewed journals, three non-peer reviewed paramedicine trade journals were included as they were deemed important to augment the written conversation through primarily editorial and commentary type articles. Collectively selecting a sample in this way provided an opportunity to balance the inclusion of sufficient information, representation, and opportunities for interpretation, with feasibility given the expansive literature base.

### Data extraction and coding

In qualitative analysis, immersion in the data is a key component of identifying emerging concepts. Through immersion, “researchers reach an overall understanding of data and also the main issues in the phenomenon under study. This understanding prepares them to focus on the most important constructs recognized and presented in data” (pg. 103) [20]. Therefore, initial identification of potential discourse began while titles and abstracts were being reviewed. A data extraction form was developed collaboratively between one author (NC) and a research associate iteratively during this process. After some piloting, the extraction form was reviewed by two other researchers (WT and IEB), resulting in a final data extraction form. As we intended to both describe the literature, then use content, thematic, and elements of discourse analysis to understand the data, the form included fields for demographic data, literature type, study results, and context.

Four research associates were trained to perform full text coding of articles; articles were split evenly, and coding was completed independently. One additional reviewer (NC) performed quality checks as the coding was completed in order to ensure consistency and trustworthiness [26]. The team met periodically to discuss progress and reconcile issues through consensus.

### Analysis

Our analysis plan included elements of content, thematic and discourse analysis as we required techniques that could both describe the data (through orderly search, selection, and extraction) and explain the meaning and context (through development and analysis of discourses). Overall, we were able to categorize each article under an emergent discourse, and then provide a narrative summary and description of the circumstances and conditions associated with the discourse (‘context’). Because of our approach, we were also able to attend to the trajectory of discourses over time.

To gain an initial understanding of the data, we employed qualitative content analysis, which permits a systematic approach while also flexibility according to the material being reviewed [21]. The purpose of the content analysis was to allow emergent and prevalent concepts to be iteratively identified [22]. We then sought to employ elements of critical discourse analysis as described above [19] which would allow us to both identify within the concepts what topics and discussions exist, how they have evolved, and how one discussion may have led to the next. In traditional discourse analysis, researchers may have “objects” or categories in mind; in this study these were not identified or outlined in advance. As with Rangel et al., we identified categories through careful reading of the literature and identified discourses around them. We conceptualized “discourses” as themes, how they were talked about and studied, as well as their trajectory.

Using this process, we were able to recognize and explore repeated concepts by defining, and stratifying them, drawing out prevalent discussions and themes, examining the context, and observing shifts in knowledge. In order to ensure trustworthiness of analysis, we worked in consultation with each other as we had during data extraction [26]. Triangulation of both methods (content, thematic, and discourse analysis) and reviewer interpretation during analysis allowed us to highlight and describe the key features of this expansive data set while moving iteratively back and forth through the data and our analysis [27].

## Results

**Fig. 1:**
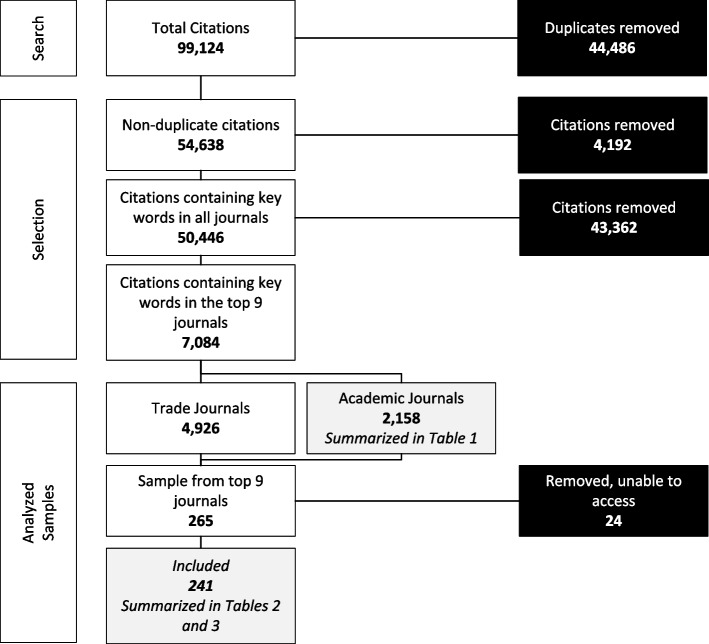
Modified PRISMA diagram of search and selection

The search strategy returned 99,124 citations, which identified 54,638 non-duplicated citations for screening (Fig. 1). After applying eligibility criteria (described above) and searching for key words, we were left with 50,446 relevant citations, 7084 relevant citations from the nine selected journals, of which 2158 were reviewed for topic and summarized in Table 1, and 241 were retrieved and included for full text review and summarized in Tables 2 and 3.

**Table 1:**
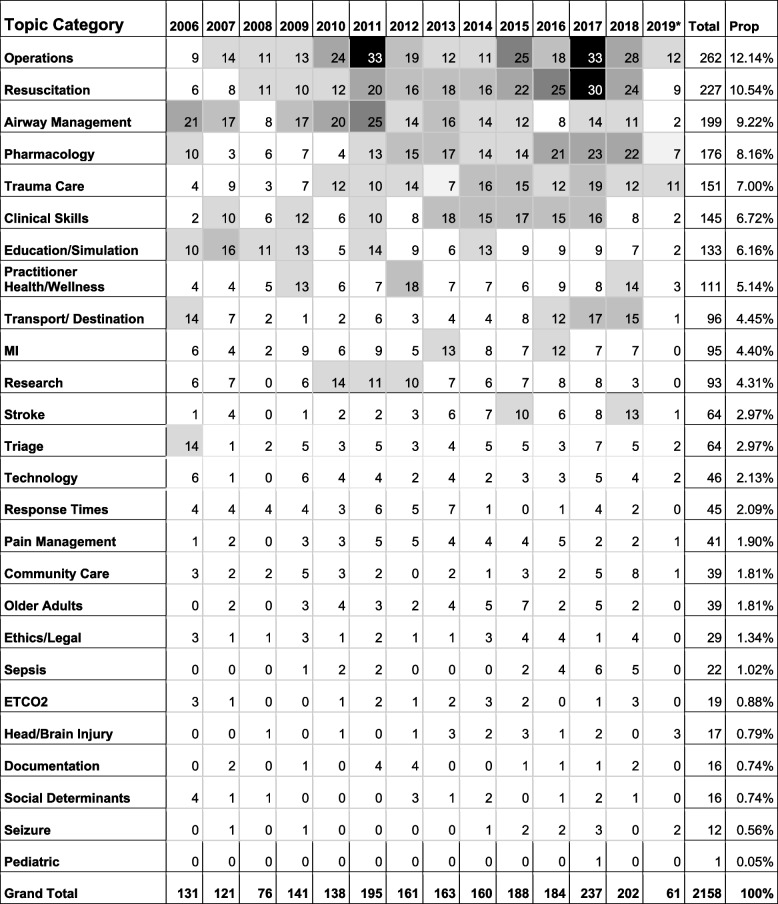
Frequency and proportion of topic categories by year for the full database (*n* = 2158). Note:  < 10;  10 to 14;  15 to 19;  20 to 24;  25 to 29;  > = 30

*2019 was not a complete year, but comprised up to and including April

**Table 2:** A narrative summary, themes, and context, for the top ten topic categories in the sample (*n* = 239; N.B., two articles could not be mapped to the top 10 topic categories and are not included in this table)

Category	# Of Articles		Themes	Context	Summary
	Relevant Articles	Sample			
**Operations:** How paramedicine functions within itself, and within a larger health system.	264	42	1. System Impact and Costs (*n* = 14) 2. Resource Utilization (*n* = 12) 3. Triage in Dispatch (*n* = 7) 4. Disaster Management (*n* = 4)	Literature in this category tended to ask questions about how paramedicine fits with other health services, including how these resources can be best used. Articles discussing “positions” (i.e., ideas about how paramedicine should operate, what roles paramedics could act in) tended to be editorial or commentary, whereas those that included clinical outcomes were more empirically focused. Additionally, approximately half of the literature came from journals specific to paramedicine, though the majority was published in trade journals. Of note, ‘Resource utilization’ literature increased in 2011/2012, with 2011 particularly focused on ambulance diversion. In 2007, and preceding the increased interest in utilization, there were articles under ‘triage in dispatch’, which studied the dispatcher’s role in ensuring appropriate use of paramedicine.	For this category, 37 of the 42 articles could be retrieved and analyzed. Of the 14 articles in the System Impact and Costs theme, 13 focused on paramedicine system planning, and described a diverse range of topics that included equipment and supplies, training, funding, staffing, alternative roles for paramedics, performance measurement, interoperability, urban and remote usage, and the optimal implementation of evidence-based guidelines [28–40]. One article focused on the impact to a single hospital from receiving air ambulance patients [41]. Of the 12 articles in the Resource Utilization theme, six articles explored system planning, which included diverse topics such as physician staffing of helicopters, planning prehospital critical care, describing specific examples of system reform, use of the system by diabetic patients, and community care in a First Nations community [42–47]. Three articles focused on emergency call management, including better understanding why people activate paramedicine systems, and management of frequent users [48–50]. Finally, one article described a program reducing hospital transportation [51], and two the use of the paramedicine system by pediatric patients [52, 53]. Of the seven articles in the Triage in Dispatch theme, five articles focused on ground ambulance dispatch, and included specific groups such as stroke and myocardial infarction and Alpha level patients, scoring the potential for a life-threatening condition, and dispatch adherence and patient outcome [54–58]. For air ambulance, the focus was on cancelling and triaging helicopter paramedicine calls [59, 60]. Of the four articles in the Disaster Management theme, three of the articles focused on system planning in specific types of disasters – blast injuries, hurricanes, and shootings [61–63]. One focused on laws and negligence in shootings [64].
**Resuscitation:** Discussion of prehospital assessment, treatment, and outcomes associated with out-of-hospital cardiac arrest and resuscitation.	229	29	1. Clinical Protocol (*n* = 9) 2. CPR (*n* = 7) 3. Survival (*n* = 7) 4. Recognition of Cardiac Arrest (*n* = 2)	Literature in this category asked questions focused on improving outcomes related to out of hospital cardiac arrest (OHCA). Early literature appears to have less focused questions, and studies were developed out of general concern for poor OHCA outcomes. Literature around 2011/2012 turned toward testing specific interventions/hypotheses. The majority of work was clinical/quantitative, however some qualitative work focused on behavior related to recognizing and acting on OHCA, for both paramedicine and bystanders. Most of the literature in this category came from academic journals related to emergency medicine, rather than academic or trade journals specific to paramedicine.	For this category 25 of the 29 articles could be retrieved and analyzed. Of the nine articles in the Clinical Protocol theme, there were two articles on non-invasive near-infrared spectroscopy (NIRS) [65, 66], two on therapeutic hypothermia [67, 68], and two on termination of resuscitation [69, 70]. There was one article respectively on double sequential external defibrillation [71], extracorporeal CPR [72], and CPR induced consciousness [73]. Of the seven articles in the CPR theme, there were two studies on bystander CPR [74, 75], and two on telephone CPR [76, 77]. There was one article respectively on CPR training technology [78], CPR during transport [79], and high-performance CPR by paramedics [80]. Of the seven articles in the Survival theme, there was a diverse range of topics with one article respectively on best practice [81], paramedic compared to physician [82], paramedic compared to bystander [83], echocardiography informed prediction [84], a national registry [85], hospital predictors [86], and research challenges [87]. Of the two articles in the recognition of cardiac arrest theme, both explore the recognition of cardiac arrest by Emergency Medical Dispatchers [88, 89].
**Airway Management:** Prehospital techniques and training for equipment and protocols for securing and managing the airway.	205	29	1. Intubation (*n* = 22) 2. Ventilation and Oxygenation (*n* = 4) 3. Airway (*n* = 3)	Articles in this category overwhelmingly focused on intubation. Many studies attached a question of safety and appropriateness to scientific investigation. The ability and necessity of paramedics to perform intubation in the field was often in question, and in studies that used clinical evaluations, paramedics were often compared to other providers such as physicians. Nearly half of literature was found in the journal *Prehospital Emergency Care*, and most of the methods were evaluation or review based. Specific topics in this category varied over time and did not appear to follow any trends, except video laryngoscopy, which began in 2011.	For this category all 29 of the articles could be retrieved and analyzed. Of the 22 articles in the Intubation theme, 12 focused on the procedure, with three primarily focused on drug assisted intubation [90–92], and two on trauma [93, 94]. The remaining seven procedure articles addressed myriad topics including intubation compared to other airway techniques, continuing competence, high-fidelity simulation, delayed sequence intubation, data, and a national committee review [95–101]. Of the seven articles on equipment, five focused on video laryngoscopy [102–106], and the remainder compared bougie use to no bougie use, and cuffed to uncuffed tubes [107, 108]. Of the three articles focusing on the provider and setting, paramedics were compared with other prehospital or hospital-based providers, one study focused on different specialties of physicians working prehospital [109–111]. Of the four articles in the Ventilation and Oxygenation theme, varied areas of focus included critical ventilation events, foreign body obstruction, non-invasive ventilation, and oxygenation guidelines [112–115]. Of the three articles in the Airway theme, articles addressed advanced or basic airway in out-of-hospital cardiac arrest, pediatric King-LT and data [116–118].
**Pharmacology:** Indication for and administration of medications for both primary and advanced care paramedics.	180	19	1. Safety, Efficacy and Effectiveness (*n* = 13) 2. Level of Paramedic Practice (*n* = 4)	In this category, the literature largely focused on the safety of medications used for sedation and pain control, and asked questions about which level of providers should administer them. Questions were most often investigated using non-experimental evaluations, and reviews, and most often appeared in academic journals. Some conversations around medication changed over time, for example naloxone was first tentatively discussed in 2013, and by 2019 it was seen as common practice for paramedics. Other conversations stayed consistent, for example early studies on ketamine focused on safety and effectiveness in prehospital care, and these questions appear to persist to present day. A number of studies looked at the level of training a paramedic needed to administer medications, asking if there was potential for those at an entry level to safely expand scope of practice. Overall, articles appear to identify a need for reliable, safe, and effective ways to use prehospital pharmacology.	For this category 17 of the 19 articles could be retrieved and analyzed. Of the 13 articles in the Safety theme, four articles addressed analgesia (two ketamine and two fentanyl) [119–122], and three opioid overdose (naloxone) [123–125]. Further articles addressed bleeding (tranexamic acid) [126], bronchospasm (oral prednisolone) [127], arrhythmia (lidocaine) [128], seizure (midazolam) [129], acute agitation (ziprasidone) [130], and heart failure (furosemide) [131]. Of the four articles in the Levels of Paramedic Practice theme, three articles addressed analgesia (2 ketamine and 1 fentanyl) [132–134], and one article hypoglycemia (oral glucose) [135].
**Trauma Care:** Assessment and treatment of traumatic injuries, as well as triage and trauma system efficiency.	155	27	1. Trauma Systems and Care (*n* = 13) 2. Hemorrhage (*n* = 8) 3. Immobilization (*n* = 6)	Literature in this category asked questions about core practices in trauma such as hemorrhage and immobilization (spinal, and other fractures) and prediction tools in an effort to improve outcomes. The majority of studies in this category were clinical and quantitative, and specific to practices seen as vital for paramedicine. Some practices, particularly those around hemorrhage, came from foundations in military medicine, which is compared to civilian paramedicine. Research related to interventions remained consistent over time, evaluating traditional interventions like spine boards, as well as innovative ones such as hemostatic dressings. The literature sources were fairly evenly split between emergency medicine journals and those specific to paramedicine, as well as between academic and trade journals.	For this category all 27 articles could be retrieved. Of the 13 articles in the trauma systems and care theme, four articles were on the management of different trauma populations, including burn victims requiring escharotomy [136], pediatric diaphragmatic injuries [137], pregnant patients [138], and minor head injuries [139]. Four articles were on triage [140–143], four were on trauma system assessment or design [144–147], and one was on prehospital levels of care [148]. Of the eight articles in the Hemorrhage theme, four articles focused on hemostatic dressings [149–152], and one each on tourniquets [153], IV infusion strategies [154], blood transfusion [155], and triage [156]. Of the six articles in the Immobilization theme, four focused on spinal immobilization [157–160], one on pelvis [161] and one on neck of femur fractures [162].
**Clinical Skills:** Foundational skills in paramedic practice, relating to the clinical assessment and care of patients.	149	30	1. Clinical Acumen (*n* = 11) 2. Specific Interventions (*n* = 11) 3. Decision Making Theory (*n* = 3) 4. Skill Description (*n* = 2)	Literature in this category commonly sought to understand paramedic decision making and clinical acumen. Up until around 2013, the focus was on thought processes around diagnosis and decision to use particular types of interventions. In more recent years, researchers have tried to identify the specific decision-making styles of paramedics in a more philosophical way. Most of the studies were qualitative in nature, with some quantitative analysis of specific interventions. Approximately half of the articles in this category were found in paramedic specific journals, however the vast majority were published in academic journals.	For this category 27 out of 30 articles could be retrieved. Of the 11 articles in the Clinical Acumen theme, three articles compared paramedic diagnosis to that of physicians [163–165], two articles described pediatric anaphylaxis [166, 167], and one each described anaphylaxis [168], pediatric resuscitation [169], sepsis [170], extracorporeal membrane oxygenation (ECMO) [171], reducing harm [172], and paramedic clinical skills [173]. Of the 11 articles in the Specific Interventions theme, three were on intraosseous access, fluid, or pressure monitoring [174–176], two were focused on intravenous access or fluid [177, 178], and one each on blood transfusion [179], spinal immobilization [180], oxygen [181], continuous positive airway pressure (CPAP) [182], tourniquets [183], and ultrasound [184]. Of the three articles in the Decision Making Theory theme, two were focused on decision making styles [185, 186], and one on decision making models [187]. Of the two articles in the Skill Description theme, one described paramedic interventions [188], and the other treating obese patients [189].
**Education/Simulation:** Paramedic training, skills development, and continuing competence.	134	28	1. Skill Based Training and Continuing Competence (*n* = 13) 2. Formative Education (*n* = 8)	Literature in this category asked broad questions about the most effective ways to educate paramedics, as well as maintain competence. Early literature tended toward training for traditional paramedicine scenarios such as mass casualty and provision of CPR, as well as general professional development. Later literature (2014 and onward) began to describe innovative training reflective of evolving paramedic role, as well as advancements in technique such as simulation. Nearly a quarter of the literature found in trade journals was in this category, and much of it was editorial, or based on survey data.	For this category 21 out of 28 articles could be retrieved and analyzed. Of the 13 articles in the Skill Based Training and Continuing Competence Theme, three focused on simulation [190–192], two on continuing education [193, 194], two on disaster training [195, 196], two on paramedic performance feedback [197, 198], and one each on research literacy [199], prescribing [200], stress inoculation [201], and training officers [202]. Of the eight articles in the Formative Education theme, three focused on education considerations [203–205], and one each on such diverse topics as assessment of practitioners [206], human factors [207], virtual reality [208], and community paramedic and critical care paramedic training [209, 210].
**Practitioner Health and Wellness:** Physical and psychological health and well-being of paramedics.	112	25	1. Fatigue (*n* = 10) 2. Psychological Wellness (*n* = 7) 3. Operational Safety (*n* = 6)	Literature in this category asked questions about both the physical and mental health of paramedics. Most of the research questions were framed in a way that put prevention of physical and psychological injury as the focus; very few focused on how practitioner injury could impact patient care. There has been a clear shift and expansion of what is meant by occupational safety. Prior to 2012, literature around practitioner health and wellness was dedicated to operational safety issues such as ambulance egress and driving. Starting in 2012, the literature has had an increased focus on discussion of fatigue, and psychological wellness of paramedics. The majority of this work was qualitative, with some editorial and commentary pieces. There were also a number of systematic reviews on the topic of fatigue, all appearing in 2018. Psychological wellness tended to appear in trade journals, where fatigue tended to appear in academic journals.	For this category 23 out of 25 articles could be retrieved and analyzed. Of the 10 articles in the Fatigue theme, there was a diverse set of topics with one article each on air ambulance crew fatigue [211], biomathematical models [212], caffeine [213], data collection instruments [214], fatigue training [215], inter-shift recovery [216], napping [217], shift pattern [218], task load [219], and sleep and safety [220]. Of the seven articles in the psychological wellness theme, three were focused on critical incidents [221–223], and one each on inclusivity (LGBTQ2) [224], mental health matters [225], rural paramedicine [226], and the “national EMS memorial” [227]. Of the six articles in the Operational Safety theme, there were two articles on paramedic fitness [228, 229], and one each on ambulance stretchers [230], lift injuries [231], scene safety [232], and firefighting [233].
**Transport/ Destination:** Effective and appropriate use of air and ground resources, and transfer of care to hospitals and other health centers.	100	16	1. Appropriate Transport (*n* = 7) 2. Offload and Diversion (*n* = 5) 3. Interfacility Transfer (*n* = 3)	Literature in this category generally asked questions about the most effective way to use paramedic resources, by looking at external influencing factors. Articles on this topic did not appear in any significant number until 2011; there were some noticeable trends. Earlier articles tended to study the use of air transport. In recent years there has been increased discussion of offload delay, from both a measurement perspective, and examining perception as reported by paramedics. The question of what constitutes a legitimate cause for ambulance transport, and what is the most effective way to transport, appears to be enduring. This category was more often seen in academic journals, particularly around the sub-theme ‘appropriate transport,’ and much of the study was based on evaluation.	For this category 15 out of 16 articles could be retrieved and analyzed. Of the seven articles in the Appropriate Transport theme, three articles focused on alternative transport, with one exploring transport to a community health centre [234], one on low-risk syncope patients [235], and one on non-urgent transports [236]. Two studies focused on transport mode, with one focused on air versus ground [237], and one on ambulance versus private car [238]. One study described an air ambulance system [239] and one was a position statement on air ambulance marketing [240]. Of the five articles in the Offload and Diversion theme, three focused on ambulance diversion [241–244], and two on offload delay [243, 245]. Of the three articles in the Interfacility Transfer theme, one focused on air versus ground transport criteria [246], one described a botulism outbreak in a regional hospital [247], and one assessed the interfacility transport process [248].
**Myocardial Infarction (MI):** Prehospital factors impacting outcomes of MI.	99	18	1. Assessment and Treatment (*n* = 16) 2. Transport (*n* = 2)	Literature in this category asked questions about the paramedic role in achieving the best outcomes for MI. Study of the topic has remained fairly consistent over time, with shift in focus every few years. Literature in 2006/2007 saw a concentrated discussion on the effectiveness of thrombolysis, whereas in 2010 and 2011 there seemed to be a “back to basics” type focus looking at interventions such as ECG and transport. From 2013 on, most literature has looked at the importance of catheter lab activations. MI literature tended to be published in academic journals, and was evenly split between journals specific to paramedicine, and those in emergency medicine; the majority of studies were empirical.	For this category all 18 articles could be retrieved and analyzed. Of the 16 articles in the Assessment and Treatment theme, four were generally focused on prehospital ECG and ST elevation MI identification and thrombolysis [249–252]. Three were focused on adverse events, with one each on acute coronary syndrome in the air medical environment [253], dextrocardia [254], and basic life support paramedic nitroglycerin use [255]. Three articles focused on patient outcomes, with one describing patients with acute chest pain [256], one percutaneous coronary intervention versus thrombolytic therapy [257], and one early versus late bleeding [258]. Three articles described paramedicine-initiated catheter lab activation [259–261]. One study focused on non-ST elevation MI [262], another on alternative lead placement [263], and another on the identification by paramedics of a rare presentation of an acute coronary syndrome [264]. Of the two articles in the Transport theme, one focused on transport time [265] and the other on ambulance diversion [266] as related to MI treatment.

**Table 3:** Summary of Methods (*n* = 241)

Method	Frequency (%)
Observational^a^	96 (40%)
Editorial/Commentary	68 (28%)
Knowledge Synthesis^b^	27 (11%)
Survey Study	15 (6%)
Intervention^c^	13 (5%)
Qualitative Research	12 (5%)
Randomized Controlled Trial	7 (3%)
Conference Proceedings	2 (< 1%)
Mixed Methods	1 (< 1%)

^a^Includes cohort, case control, and case series

^b^Includes all types of literature reviews (e.g., systematic reviews, scoping reviews, narrative reviews, etc.)

^d^Includes pre/post implementation designs that are not randomized

The six peer reviewed journals included in the review were: The Journal of The American Medical Association, Prehospital Emergency Care, Academic Emergency Medicine, Emergency Medicine Journal, the Canadian Journal of Emergency Medicine, and BMC Emergency Medicine. The three trade journals selected for the review were: Journal of Emergency Medical Services, Journal of Paramedic Practice, and Canadian Paramedicine.

The categorization of peer reviewed citations from the 2158 relevant citations is summarized in Table 1. This table describes the frequency and publication year for the 26 identified topic categories. Briefly, the top three categories were operations (*n* = 262, 12.1%), resuscitation (*n* = 227, 10.5%) and airway management (*n* = 199, 9.2%). Certain topics (e.g., Operations, Resuscitation, and Pharmacology) appear to have more attention in recent years, whereas others (e.g., Airway Management, Education and Simulation, and Research) saw publication activity decline. The top 10 categories (Operations, Resuscitation, Airway Management, Pharmacology, Trauma Care, Clinical Skills, Education/Simulation, Practitioner Health and Wellness, Transport/Destination, and Myocardial Infarction) accounted for 74% of the 2158 citations. Table 2 provides a full narrative summary and description of context, based on the final sample of 241 citations. Trade journal citations were successfully mapped to the top 10 peer reviewed topic categories in all cases, except two. These two articles described the development of the paramedic profession (one describing a strategic plan for a college of paramedicine, and the other describing self-regulation). Each category presented in Table 2 was defined and further described through theming, resulting in between two and four themes per category (*n* = 30 total themes). For example, the Operations category was defined as “How paramedicine functions within itself, and within a larger health system”; there were four themes identified including System Impact and Costs, Resource Utilization, Triage in Dispatch, and Disaster Management. Additional detail is also provided on the context of the articles in each category, and a summary of those articles in terms of detailed study subject. We reflect further on the context of these findings in our discussion.

Table 3 describes the article types and research methods that were used in the final sample of 241 citations. The most common was observational research approaches (*n* = 96, 40%), which included cohort, case control, and case series. The second most common was editorial/commentary (*n* = 68, 28%), followed by knowledge synthesis activities (*n* = 27, 11%), which included systematic reviews, scoping reviews, etc.

## Discussion

In this study we attempted to reveal the academic pursuits and research conversations the paramedicine community has focused on by exploring the literature since 2006. Trends, and frequency related to what has been published in recent history provide an opportunity to reflect on what has been deemed important to those aiming to advance paramedicine and where intended future directions for the profession may be attended to or not. Our results suggest: 1) a relatively narrow topic focus that does not entirely align with the priorities in aspirational and direction setting reports, given the majority of research has concentrated on general operational activities and specific clinical conditions and interventions (e.g., resuscitation, airway management, etc.); 2) a limited methodological (and possibly philosophical) focus, given that many were observational studies and editorial or commentary; and 3) a variety of observed trajectories of academic attention, indicating where research pursuits and priorities are shifting, and where confidence in the profession is situated. We discuss each in turn with implications for the profession to consider.

### Scope of literature

The literature included in this study presents a narrow scope of primarily clinically focused topics such as resuscitation, airway management, and pharmacology. We also saw numerous articles that discussed how operations support clinical care. This appears to align, at least superficially, with priorities identified in the Canadian National Research Agenda, where 21 out of the 36 priority areas requiring additional or increased research attention fit into these topics [4, 10]. However, this finding does not directly support many priorities presented in a number of seminal reports published over the years that have endeavored to set a strategy for the future of paramedicine [6–9, 20], including the recently published principles document in Canada [14]. These seminal reports tended to focus on issues that highlighted the adaptability and advancement of paramedicine, which included concepts such as how paramedicine can provide novel approaches to primary and community care, how to improve quality of emergency services using evidence, how to develop and diversify the paramedicine workforce, how to create safe and sustainable workplaces, how to integrate paramedic care in the health system, and how to support a change in culture that will lead to new directions and models of care. Some of the literature topics can indeed be mapped to these aspirational and direction setting reports (e.g., a small amount of literature in the Operations category discussed paramedic system impact, and literature in some clinical areas that looked to improve interventions). However, this leaves other areas under-represented. These may include for example, ways in which paramedicine can leverage its adaptability and develop models, practitioners, and leaders that support an autonomous profession providing health care in a variety of settings, and meaningfully contributing to the health and social well-being of communities.

### Scope of article types and methods

In addition to a narrow focus of topics, we also observed a narrow methodological focus. The primarily observational study methods, as opposed to methods that provide explanations (e.g., qualitative and mixed methods), suggested more of a focus on the “what” rather than the “why”. The attention on observational studies highlights two insights: first, the feasibility of certain types of study methods may be a challenge in paramedicine. For example, randomized control trials are complex and expensive to implement in the relatively austere environment in which paramedicine is practiced. Additionally, unlike many areas of medicine, there is a limited evidence base making it difficult to determine the ethical appropriateness of randomizing interventions in paramedicine care. Second, the methods likely reflect the philosophical positions informing research contributions and the research questions asked. As stated above, the types of clinical questions studied generally lend themselves to quantitative methods or perhaps positivist or post-positivist approaches. However, as mentioned in the discussion of the seminal aspirational and direction setting reports, most often written by paramedic leaders rather than academic researchers, future research questions may tend toward topics related to the development of the profession (e.g., development and diversification of the paramedic workforce, creation of safe and sustainable workplaces, etc.); meaning we may see the use of broader methodologies (e.g., qualitative and mixed methods) and philosophical lenses as time goes on.

### Trajectory of research and conversations

Evolution of research questions and methods, as well as shifts seen within topics, help us gain insight into the trajectory of the paramedic profession itself. The literature highlights areas where the evolution of paramedicine is evident, areas where scope of practice is uncertain, and areas that aim to improve skills that have historically been considered core to paramedic clinical practice. Some of the topics in the literature clearly indicate where the conversation has evolved. An example is in the use of naloxone in paramedicine. In 2013, we see validation of criteria for the use of naloxone by paramedics and a conclusion that the drug is underutilized in the treatment of drug-related altered mental status [123]. In 2014, the evidence evolves to suggest that the scope and route of administration for naloxone must be cautiously considered to address the proliferation of opioid overdose [124]. Finally in 2019, a systematic review further progresses the discussion to ask if patients treated with naloxone can be safely left on scene [125]. There are also topics that highlight uncertainty around the scope of paramedicine care. This research tends to persistently focus on questions of whether paramedics can safely administer a treatment. For example, endotracheal intubation (ETI) has long been held as the gold standard for airway management. The language in some articles related to paramedicine suggest the authors remain unsure whether paramedics should be providing the intervention [93, 267, 268]. Finally, and in contrast to the topics where uncertainty existed, there are topics that continue to advance traditional paramedic skills. Studies that investigated subjects like trauma care and resuscitation tended to focus on new and innovative techniques, or improving existing techniques, with no questioning of whether paramedics should perform the skill (e.g., spinal immobilization) [158–160]. To be clear, we are not suggesting that interventions administered by paramedics should not be scrutinized, in fact it is critical that all health professions study what they do and how they do it. Our observation is that there were certain types of interventions where questions were persistently more focused on asking if a paramedic should do something, and others where the focus was around how it could be done better.

## Implications

Paramedicine is full of potential, and uniquely positioned in the health care system to provide community care that ranges from emergency response to chronic disease management and palliative care. This review highlights that while paramedicine is in a state of rapid change as evidenced from aspirational and visioning documents, the evidence and knowledge generation to inform these changes may not be keeping pace as evidenced by the narrow topic and methodological focus described in this review. If we are to build evidence informed practice and systems in paramedicine, which was one of the principles identified by Canadian paramedicine stakeholders [14], the scholarly paramedicine community must connect aspirational and visioning documents with evidence that will inform the vision, and translation of knowledge to achieve the vision. This may lead stakeholders to consider the capacity that is presently available to create knowledge, and whether it is sufficiently resourced and focused to support paramedicine into the future. For example, do we have sufficient numbers of PhD trained researchers to support the research enterprise, and do we have a robust foundation of leadership science to move paramedicine into the future? It may also lead to questions such as what research questions are being asked, why are these questions being asked, and who is asking them?

To meet this potential, researchers can leverage the existing areas of strength (e.g., evidence from topics that have been studied extensively, and activities that have been shown to be effective and efficient) and broaden both focus and capacity. This could be achieved by looking to aspirational and direction setting reports to identify organizational and professional priorities, which may require refreshing extant documents (e.g., Canadian National EMS Research Agenda, PCC visioning document, National Occupational Competency Profile, etc.), and expanding the topic and methodological foci by creating communities of practice that include diverse skill sets and expertise. Through knowledge of our current evidence, careful examination of how paramedicine is moving forward, and purposeful collaboration, we can ensure that the evidence base for paramedicine supports all aspects of this adaptable and innovative profession and systems.

## Limitations

The database was extensive (over 50,000 citations), which required distilling to a representative sample, risking some loss of information and introduction of selection bias. While our intention was to be reflective and stimulate discussion on what is being attended, rather than an exact map of every publication in paramedicine, it is important to consider how the sample was generated. For example, we recognize that there are some issues with Journal Impact Factor [269]. We de-emphasized the weight of the impact factor by emphasizing the proportion of paramedicine articles published by the journal, as well as the country of publication. The selection of largely North American journals may have captured Directive systems, where there is strong medical oversight and control of paramedics, compared to Professionally Autonomous systems, where there has been focused development of the role of paramedicine [270, 271]. This may have influenced the content of those journals. We did not include the Medical Subject Heading (MeSH) term “emergency medical technician” which may have systematically excluded articles related to basic life support care in the US. We feel the magnitude of this exclusion is small (i.e., approximately 60% of citations that include the “emergency medical technician” term were retrieved using the search strategy), and would likely not have influenced the reported topics, though it could conceivably have added to the level of detail discussed in certain categories.

The data required some degree of subjectivity, but wherever possible this was discussed thoroughly between research team members to ensure consistency and trustworthiness. For example, in the Operations category, extensive discussion ensued over what this meant and how to ensure consistent leveling of this category with other categories that were similar (e.g., response time, etc.). Additionally, it should be noted that we were not attempting to infer importance of the topics, but to reflect the breadth of topics that were retrieved. We approached this study as readers and consumers of the information and believe our process of having more than one reviewer per article provided assurances that our interpretations as knowledge users were appropriate. We did not consider the quality of the evidence or what direction the evidence was pointing: instead, we focused on what questions and topics were being attended to in the literature, and how and when it was studied.

## Conclusions

Included articles suggest a relatively narrow topic focus, a limited methodological (and possibly philosophical) focus, and a variety of observed trajectories of academic attention, indicating where research pursuits and priorities are shifting, and where confidence in the profession is situated. We have highlighted that the academic focus may require an alignment with aspirational and direction setting documents aimed at developing paramedicine. This review when placed in historical context may be a snapshot of scholarly activity that reflects a young medically directed profession and systems focusing on a few high acuity conditions, with aspirations of professional autonomy in a supportive system collaborating with medicine to provide health care in a variety of settings and contributing to the health and social well-being of communities.

## Data Availability

The datasets used and/or analysed during the current study are available from the corresponding author on reasonable request.

## Abbreviations

EMS: Emergency Medical Services
MeSH: Medical Subject Heading
PCC: Paramedic Chiefs of Canada

## Footnotes

1 We are defining paramedicine in the broadest possible sense, which includes multiple specialized services and professions within the broader system, including communications officers (i.e., emergency and community paramedic call taking, and dispatch and strategic organization of paramedic units), medical consultation, supply chain, education, quality improvement, data analytics, infection prevention and control, employee programs (i.e., peer and professional support), specialized operational teams (e.g., tactical, CBRNE, air ambulance, etc.), and research.

## References

[1] Williams B, Beovich B, Olaussen A (2021). The definition of paramedicine: an international delphi study. J Multidiscip Healthc.

[2] Eaton G (2019). Paramedic. noun. Br Paramed J.

[3] Callaham M (1997). Quantifying the scanty science of prehospital emergency care. Ann Emerg Med.

[4] Jensen JL, Bigham BL, Blanchard IE, Dainty KN, Socha D, Carter A (2013). The Canadian National EMS research agenda: a mixed methods consensus study. CJEM..

[5] Tavares W, Bowles R, Donelon B (2016). Informing a Canadian paramedic profile: framing concepts, roles and crosscutting themes. BMC Health Serv Res.

[6] Deakin CD, King P, Thompson F, Deakin CD, King P, Thompson F (2009). Prehospital advanced airway management by ambulance technicians and paramedics: is clinical practice sufficient to maintain skills?. Emerg Med J.

[7] Bigham BL, Kennedy SM, Drennan I, Morrison LJ (2013). Expanding paramedic scope of practice in the community: a systematic review of the literature. Prehosp Emerg Care.

[8] Thurman WA, Moczygemba LR, Tormey K, Hudzik A, Welton-Arndt L, Okoh C (2021). A scoping review of community paramedicine: evidence and implications for interprofessional practice. J Interprof Care.

[9] Beovich B, Olaussen A, Williams B (2021). A bibliometric analysis of paramedicine publications using the Scopus database: 2010–2019. Int Emerg Nurs.

[10] J J (2013). Canadian national research agenda.

[11] Jensen JL, Blanchard IE, Bigham BL, Carter A, Brown R, Socha D (2015). The Canadian National EMS research agenda: impact and feasibility of implementation of previously generated recommendations. CJEM..

[12] Jensen JL, Blanchard IE, Bigham BL, Dainty KN, Socha D, Carter A (2011). Methodology for the development of a Canadian national EMS research agenda. BMC Emerg Med.

[13] Dainty KN, Jensen JL, Bigham BL, Blanchard IE, Brown LH, Carter AJ (2013). Developing a Canadian emergency medical services research agenda: a baseline study of stakeholder opinions. Can J Emerg Med.

[14] Tavares W, Allana A, Beaune L, Weiss D, Blanchard I. Principles to Guide the Future of Paramedicine in Canada. Prehosp Emerg Care. 2022;26(5):728–38. 10.1080/10903127.2021.1965680 34376112

[15] Bank J (2014). Canadian paramedic services standards report: a strategic planning report.

[16] Olaussen A, Beovich B, Williams B (2021). Top 100 cited paramedicine papers: a bibliometric study. Emerg Med Australas.

[17] Rangel JC, Cartmill C, Kuper A, Martimianakis MA, Whitehead CR (2016). Setting the standard: medical education’s first 50 years. Med Educ.

[18] Gee JP (2004). Discourse analysis: what makes it critical? An introduction to critical discourse analysis in education.

[19] Rogers R (2004). An introduction to critical discourse analysis in education.

[20] Vaismoradi M, Jones J, Turunen H, Snelgrove S (2016). Theme development in qualitative content analysis and thematic analysis.

[21] Mayring P (2004). Qualitative content analysis. Companion Qualit Res.

[22] Hsieh H-F, Shannon SE (2005). Three approaches to qualitative content analysis. Qual Health Res.

[23] Ludwig G, Leadership sector. (2009). Media ‘trauma’: new NBC series gives managers something to think about. JEMS. J Emerg Med Serv.

[24] Burgess S, Smith E, Piper S, Archer F (2010). The development of an updated prehospital search filter for the Cochrane library: prehospital search filter version 2.0. J Emerg Prim Health C.

[25] Olaussen A, Semple W, Oteir A, Todd P, Williams B (2017). Paramedic literature search filters: optimised for clinicians and academics. BMC Med Inform Decis Mak.

[26] Elo S, Kääriäinen M, Kanste O, Pölkki T, Utriainen K, Kyngäs H (2014). Qualitative content analysis: a focus on trustworthiness. SAGE Open.

[27] Nowell LS, Norris JM, White DE, Moules NJ (2017). Thematic analysis: striving to meet the trustworthiness criteria. Int J Qual Methods.

[28] Armstrong RJ (2014). EMS: It’s not all about response time. Can Paramed.

[29] Flanagan J (2014). Joint emergency services interoperability programme: working together saving lives. J Paramed Pract.

[30] Gunderson M, Guest commentary (2009). The value quotient: looking at the combined effects of quality & cost. JEMS.

[31] Heightman AJ (2018). From the editor. Change on the horizon: EMS agencies must learn to adapt to new challenges. JEMS.

[32] Heightman AJ (2010). The challenges of clinical leadership. JEMS. J Emerg Med Serv.

[33] Heightman AJ (2016). Focused & forward thinking. JEMS.

[34] Jason U (2011). Ambulance driver. JEMS..

[35] Ludwig G (2009). Leadership sector. No bailout for EMS: running your system on less without running it into the ground. JEMS.

[36] National Association of EMS Physicians, American College of Emergency Physicians, American College of Surgeons Committee on Trauma. Equipment for ambulances: a joint statement from the National Association of EMS Physicians, the American College of Emergency Physicians, and the American College of Surgeons Committee on Trauma. Prehosp Emerg Care. 2007;11(3):326–9. 10.1080/10903120701348297 17613908

[37] Anonymous (2016). National Characteristics of emergency medical services in frontier and remote areas. Prehosp Emerg Care.

[38] Fishe JN, Crowe RP, Cash RE, Nudell NG, Martin-Gill C, Richards CT (2018). Implementing prehospital evidence-based guidelines: a systematic literature review. Prehosp Emerg Care.

[39] Patterson PD, Jones CB, Hubble MW, Carr M, Weaver MD, Engberg J (2010). The longitudinal study of turnover and the cost of turnover in emergency medical services. Prehosp Emerg Care.

[40] Whalen S, Goldstein J, Urquhart R, Carter A (2017). Paramedics perception of working in Nova Scotia's collaborative emergency centres. Can J Emerg Med.

[41] Jenkinson E, Currie A, Bleetman A. The impact of a new regional air ambulance service on a large general hospital. Emerg Med J. 2006 May 1;23(5):368–71. 10.1136/emj.2005.027045 PMC2564086 16627838

[42] Arya V, Carter WW, Robertson SM (2010). The role of clinical pharmacology in supporting the emergency use authorization of an unapproved anti-influenza drug, peramivir. Clin Pharmacol Ther.

[43] Mackenzie R, Steel A, French J, Wharton R, Lewis S, Bates A (2009). Views regarding the provision of prehospital critical care in the UK. Emerg Med J.

[44] Benoit SR, Kahn HS, Geller AI, Budnitz DS, Mann NC, Dai M (2018). Diabetes-related emergency medical service activations in 23 states, United States 2015. Prehosp Emerg Care.

[45] Pearce EA, Cody MD, White Iv CC (2018). Community care: EMS-based urgent care flourishes on the Ramah (N.M.) Navajo reservation. JEMS.

[46] Wong HT, Lai PC (2012). Weather inference and daily demand for emergency ambulance services. Emerg Med J.

[47] Heightman AJ (2012). The crews are key!: specialized EMS team deployment yields best results. JEMS..

[48] Coleman P, Turner J (2011). Patient priorities and decision-making about using 999 EMS? A review of the literature. Emerg Med J.

[49] Tadros AS, Castillo EM, Chan TC, Jensen AM, Patel E, Watts K (2012). Effects of an emergency medical services-based resource access program on frequent users of health services. Prehosp Emerg Care.

[50] Smith DP, McNally A (2014). Delivering enhanced safety, productivity and experience: early results from a frequent caller management system. J Paramed Pract.

[51] Toyoda Y, Mastuo Y, Tanaka H, Fujiwara H, Takatorige T, Iso H (2007). Prehospital score for acute disease: a community-based observational study in Japan. BMC Emerg Med.

[52] Shah MN, Cushman JT, Davis CO, Bazarian JJ, Auinger P, Friedman B (2008). The epidemiology of emergency medical services use by children: an analysis of the national hospital ambulatory medical care survey. Prehosp Emerg Care.

[53] Bober JG (2016). Pediatric ambulance use in the United States: the role of health insurance. Acad Emerg Med.

[54] Cone DC, Galante N, MacMillan DS (2008). Can emergency medical dispatch systems safely reduce first-responder call volume?. Prehosp Emerg Care.

[55] Ellensen EN, Wisborg T, Hunskaar S, Zakariassen E (2016). Dispatch guideline adherence and response interval-a study of emergency medical calls in Norway. BMC Emerg Med.

[56] Hinchey P, Myers B, Zalkin J, Lewis R, Garner D (2007). Low acuity EMS dispatch criteria can reliably identify patients without high-acuity illness or injury. Prehosp Emerg Care.

[57] Ohshige K, Kawakami C, Mizushima S, Moriwaki Y, Suzuki N (2009). Evaluation of an algorithm for estimating a patient’s life threat risk from an ambulance call. BMC Emerg Med.

[58] Evenson KR, Brice JH, Rosamond WD, Lellis JC, Christian JB, Morris DL (2007). Statewide survey of 911 communication centers on acute stroke and myocardial infarction. Prehosp Emerg Care.

[59] Giannakopoulos GF, Bloemers FW, Lubbers WD, Christiaans HMT, van Exter P, De Lange-de Klerk ESM (2012). Criteria for cancelling helicopter emergency medical services (HEMS) dispatches. Emerg Med J.

[60] Wilmer I, Chalk G, Davies GE, Weaver AE, Lockey DJ (2015). Air ambulance tasking: mechanism of injury, telephone interrogation or ambulance crew assessment?. Emerg Med J.

[61] Lerner EB, O'Connor RE, Schwartz R, Brinsfield K, Ashkenazi I, Degutis LC (2007). Blast-related injuries from terrorism: an international perspective. Prehosp Emerg Care.

[62] Clayton L, Walsh B, Troncoso A, Allegra J (2013). Impact of hurricane sandy on the presenting complaints of prehospital advanced life support patients. Acad Emerg Med.

[63] Klassen AB, Marshall M, Dai M, Mann NC, Sztajnkrycer MD (2019). Emergency medical services response to mass shooting and active shooter incidents, United States, 2014-2015. Prehosp Emerg Care.

[64] Wirth SR (2018). Care under fire: negligence principles in mass shootings. JEMS.

[65] Drennan I, Gilgan J, Goncharenko K, Lin S (2018). Paramedics using near-infrared spectroscopy in out-of-hospital cardiac arrest: a feasibility study. Can J Emerg Med.

[66] Frisch A, Suffoletto BP, Frank R, Martin-Gill C, Menegazzi JJ (2012). Potential utility of near-infrared spectroscopy in out-of-hospital cardiac arrest: an illustrative case series. Prehosp Emerg Care.

[67] Higgins GL, Kendall KM, Buyers ES, Baumann MR (2011). Can therapeutic hypothermia for post-cardiac arrest patients be successfully initiated in the prehospital environment by the clinical providers of a rural air medical service?. Acad Emerg Med.

[68] Kim F, Nichol G, Maynard C, Hallstrom A, Kudenchuk PJ, Rea T (2014). Effect of prehospital induction of mild hypothermia on survival and neurological status among adults with cardiac arrest a randomized clinical trial. JAMA..

[69] Cheong RW, Li H, Doctor NE, Ng YY, Goh ES, Leong BSH (2016). Termination of resuscitation rules to predict neurological outcomes in out-of-hospital cardiac arrest for an intermediate life support prehospital system. Prehosp Emerg Care.

[70] Chiang WC, Huang YS, Hsu SH, Chang AM, PCI K, Wang HC (2017). Performance of a simplified termination of resuscitation rule for adult traumatic cardiopulmonary arrest in the prehospital setting. Emerg Med J.

[71] Beck LR, Ostermayer DG, Ponce JN, Srinivasan S, Wang HE. Effectiveness of prehospital dual sequential defibrillation for refractory ventricular fibrillation and ventricular tachycardia cardiac arrest. Prehosp Emerg Care. 2019;23(5):1–6. 10.1080/10903127.2019.1584256 30773983

[72] Grunau B, Reynolds J, Scheuermeyer F, Stenstom R, Stub D, Pennington S (2016). Relationship between time-to-ROSC and survival in out-of-hospital cardiac arrest ECPR candidates: when is the best time to consider transport to hospital?. Prehosp Emerg Care.

[73] Pound J, Verbeek PR, Cheskes S (2017). CPR induced consciousness during out-of-hospital cardiac arrest: a case report on an emerging phenomenon. Prehosp Emerg Care.

[74] Burla M, Amen A, Otero R, Swor R (2018). Do we really know who receives bystander cardiopulmonary resuscitation prior to emergency medical services arrival?. Acad Emerg Med.

[75] Bouland AJ, Halliday MH, Comer AC, Levy MJ, Seaman KG, Lawner BJ (2017). Evaluating barriers to bystander CPR among laypersons before and after compression-only CPR training. Prehosp Emerg Care.

[76] Ong MEH, Shin SD, Tanaka H, Ma MH-M, Nishiuchi T, Lee EJ (2015). Rationale, methodology, and implementation of a dispatcher-assisted cardiopulmonary resuscitation trial in the Asia-Pacific (Pan-Asian resuscitation outcomes study phase 2). Prehosp Emerg Care.

[77] Ateyyah KA, Cady CE, Poltrock JT, Pirrallo RG (2015). A novel use of a metronome in dispatcher-assisted cardiopulmonary resuscitation. Prehosp Emerg Care.

[78] Clinkard D, Stuart K, Stuart L, Fichtinger G, Ungi T (2015). Improving CPR training by tracking: a free open-source computer program to collect Laerdal SimMan 3G CPR performance data. Prehosp Emerg Care.

[79] Hock Ong ME, Shin SD, Sung SS, Tanaka H, Huei-Ming M, Song KJ (2013). Recommendations on ambulance cardiopulmonary resuscitation in basic life support systems. Prehosp Emerg Care.

[80] Bobrow BJ, Clark LL, Ewy GA, Chikani V, Sanders AB, Berg RA (2008). Minimally interrupted cardiac resuscitation by emergency medical services for out-of-hospital cardiac arrest. JAMA.

[81] Lyon R, Sinclair N, Henderson C (2010). Improving survival from out-of-hospital cardiac arrest. J Paramed Pract.

[82] Fukuda T, Ohashi-Fukuda N, Kondo Y, Hayashida K, Kukita I (2018). Association of Prehospital Advanced Life Support by physician with survival after out-of-hospital cardiac arrest with blunt trauma following traffic collisions: Japanese registry-based study. JAMA Surg.

[83] Kaji AH, Hanif AM, Niemann JT (2011). Advanced rescuer- versus citizen-witnessed cardiac arrest: is there a difference in outcome?. Prehosp Emerg Care.

[84] Aichinger G, Zechner PM, Prause G, Sacherer F, Wildner G, Anderson CL (2012). Cardiac movement identified on prehospital echocardiography predicts outcome in cardiac arrest patients. Prehosp Emerg Care.

[85] Masterson S, Cullinan J, McNally B, Deasy C, Murphy A, Wright P (2016). Out-of-hospital cardiac arrest attended by ambulance services in Ireland: first 2 years’ results from a nationwide registry. Emerg Med J.

[86] Liu JM, Yang Q, Pirrallo RG, Klein JP, Aufderheide TP (2008). Hospital variability of out-of-hospital cardiac arrest survival. Prehosp Emerg Care.

[87] Lyon RM, Egan G, Gowens P, Andrews P, Clegg G (2010). Issues around conducting prehospital research on out-of-hospital cardiac arrest: lessons from the TOPCAT study. Emerg Med J.

[88] Vaillancourt C, Kasaboski A, Charette M, Calder L, Boyle L, Nakao S (2017). Implementation of an educational program to improve the cardiac arrest diagnostic accuracy of ambulance communication officers: a concurrent control before-after study. Can J Emerg Med.

[89] Jensen JL, Vaillancourt C, Tweedle J, Kasaboski A, Charette M, Grimshaw J (2012). Factors associated with the successful recognition of abnormal breathing and cardiac arrest by ambulance communications officers: a qualitative iterative survey. Prehosp Emerg Care.

[90] Wang HE, Davis DP, O'Connor RE, Domeier RM (2006). Drug-assisted intubation in the prehospital setting (resource document to NAEMSP position statement). Prehosp Emerg Care.

[91] Cole CD, Wang HE, Abo BN, Yealy DM (2006). Drug-assisted effects on protective airway reflexes during out-of-hospital endotracheal intubation (preliminary report). Prehosp Emerg Care.

[92] MacDonald A, MacDonald RD, Lee JS (2012). Factors affecting success of prehospital intubation in an air and land critical care transport service: results of a multivariate analysis. Acad Emerg Med.

[93] French J, Steel A, Clements R, Lewis S, Wilson M, Teasdale B (2006). Best BETS: a call for scrutiny. Emerg Med J.

[94] Evans CCD, Brison RJ, Howes D, Stiell IG, Pickett W (2013). Prehospital non-drug assisted intubation for adult trauma patients with a Glasgow coma score less than 9. Emerg Med J.

[95] Jackson M (2010). Joint Royal College Ambulance Liaison Committee Airway Working Group commentary. Emerg Med J.

[96] Le Clerc S, Henning J, Masud S (2008). Announcing the launch of a new web-based prehospital rapid sequence induction log for doctors.

[97] Waack J, Shepherd M, Andrew E, Bernard S, Smith K (2018). Delayed sequence intubation by intensive care flight paramedics in Victoria. Austral Prehosp Emerg Care.

[98] Anonymous (2010). Abstracts of the 2010 NAEMSP scientific assembly. January 7-9, 2010. Phoenix, Arizona, USA. Prehosp Emerg Care.

[99] Ward S, Studnek JR, Vandeventer S (2010). Improving prehospital endotracheal intubation psychomotor skills using high fidelity human patient simulators. Acad Emerg Med.

[100] Jensen JL, Cheung KW, Tallon JM, Travers AH (2010). Comparison of tracheal intubation and alternative airway techniques performed in the prehospital setting by paramedics: a systematic review. CJEM: Canadian. J Emerg Med.

[101] Hasegawa K, Hiraide A, Brown DFM (2013). Prehospital airway management for out-of-hospital cardiac arrest - reply. JAMA..

[102] Haddow P (2011). What is the scope for new devices for tracheal intubation?. J Paramed Pract.

[103] Guyette FX, Farrell K, Carlson JN, Callaway CW, Phrampus P (2013). Comparison of video laryngoscopy and direct laryngoscopy in a critical care transport service. Prehosp Emerg Care.

[104] Nowicki TA, Suozzi JC, Dziedzic M, Kamin R, Donahue S, Robinson K (2009). Comparison of use of the the [sic] Airtraq with direct laryngoscopy by paramedics in the simulated airway. Prehosp Emerg Care.

[105] Savino PB, Reichelderfer S, Mercer MP, Wang RC, Sporer KA (2017). Direct versus video laryngoscopy for prehospital intubation: a systematic review and meta-analysis. Acad Emerg Med.

[106] Aziz M, Dillman D, Kirsch JR, Brambrink A (2009). Video laryngoscopy with the Macintosh video laryngoscope in simulated prehospital scenarios by paramedic students. Prehosp Emerg Care.

[107] Messa MJ, Kupas DF, Dunham DL (2011). Comparison of bougie-assisted intubation with traditional endotracheal intubation in a simulated difficult airway. Prehosp Emerg Care.

[108] Mason AM (2007). Counting angels.

[109] Reichert RJ, Gothard M, Gothard MD, Schwartz HP, Bigham MT (2018). Intubation success in critical care transport: a multicenter study. Prehosp Emerg Care.

[110] Fullerton JN, Roberts KJ, Wyse M (2011). Should non-anaesthetists perform pre-hospital rapid sequence induction? An observational study. Emerg Med J.

[111] Fouche PF, Stein C, Simpson P, Carlson JN, Doi SA (2017). Nonphysician out-of-hospital rapid sequence intubation success and adverse events: a systematic review and meta-analysis. Ann Emerg Med.

[112] Soroudi A, Shipp HE, Stepanski BM, Ray LU, Murrin PA, Chan TC (2007). Adult foreign body airway obstruction in the prehospital setting. Prehosp Emerg Care.

[113] Willmore A, Dionne R, Maloney J, Ouston E, Stiell IG (2013). A before-after study to evaluate the effectiveness and use-fulness of prehospital noninvasive ventilation in an urban setting. Can J Emerg Med.

[114] Ricketts W (2012). Guideline alert: British thoracic society emergency oxygen use in adult patients. J Paramed Pract.

[115] Singh JM, Ferguson ND, MacDonald RD, Stewart TE, Schull MJ (2009). Ventilation practices and critical events during transport of ventilated patients outside of hospital: a retrospective cohort study. Prehosp Emerg Care.

[116] Silvestri S, Card K, McCoy S, Nelson J, Redden D, Papa L (2013). Assessing prehospital airway management using the florida EMS tracking and reporting system (EMSTARS). Acad Emerg Med.

[117] Ritter SC, Guyette FX (2011). Prehospital pediatric king lt-d use: a pilot study. Prehosp Emerg Care.

[118] Fouche PF, Simpson PM, Bendall J, Thomas RE, Cone DC, Doi SAR (2014). Airways in out-of-hospital cardiac arrest: systematic review and meta-analysis. Prehosp Emerg Care.

[119] Bredmose PP, Lockey DJ, Grier G, Watts B, Davies G (2009). Pre-hospital use of ketamine for analgesia and procedural sedation. Emerg Med J.

[120] Byyny R, Soriya G, Colwell C, Liao MM, Haukoos JS, McVaney K (2010). Safety of prehospital single-dose fentanyl in adult trauma patients. Acad Emerg Med.

[121] Keseg D, Cortez E, Rund D, Caterino J (2015). The use of prehospital ketamine for control of agitation in a metropolitan firefighter-based EMS system. Prehosp Emerg Care.

[122] Lebin JA, Akhavan AR, Hippe DS, Gittinger MH, Pasic J, McCoy AM, et al. Psychiatric outcomes of patients with severe agitation following administration of prehospital ketamine. Acad Emerg Med. 2019;26(8):889–96. 10.1111/acem.13725 30873690

[123] Friedman MS, Manini AF (2013). Validation of criteria to guide prehospital antidote administration for drug overdoses. Acad Emerg Med.

[124] Zuckerman M, Weisberg SN, Boyer EW (2014). Pitfalls of intranasal naloxone. Prehosp Emerg Care.

[125] Greene JA, Deveau BJ, Dol JS, Butler MB (2019). Incidence of mortality due to rebound toxicity after ‘treat and release’ practices in prehospital opioid overdose care: a systematic review. Emerg Med J.

[126] de Guzman R, Polykratis IA, Sondeen JL, Darlington DN, Cap AP, Dubick MA (2013). Stability of tranexamic acid after 12-week storage at temperatures from −20°C to 50°C. Prehosp Emerg Care.

[127] Silverman R, Lai P, Albanese J, Isaacs D, Avarello J, Foltin G (2018). Prehospital administration of steroids for pediatric asthma is influenced by illness appearance. Acad Emerg Med.

[128] Millin M, Kim S, Schmidt T, Daya M, Fujisaki B (2006). Intermittent bolus dosing of lidocaine in emergency medical services - an alternative to bolus followed by a drip. Prehosp Emerg Care.

[129] Spano SJ, Shalit M, Stroh G (2013). Intranasal midazolam is a viable alternative to intravenous midazolam for prehospital seizure. Acad Emerg Med.

[130] Hunter C, Silvestri S, Ralls GA, Stone M, Walker A, Miller S (2017). Intramuscular ziprasidone for acute agitation in prehospital patients. Acad Emerg Med.

[131] Pan A, Shell IG, Dionne R, Maloney J (2012). The use of furosemide in the prehospital setting for the treatment of heart failure. Can J Emerg Med.

[132] Cousins R, Anderson D, Dehnisch F, Brown A, McKay S, Glassman ES (2017). It’s time for EMS to administer ketamine analgesia. Prehosp Emerg Care.

[133] Lebon J, Fournier F, Begin F, Hebert D, Fleet R, Foldes-Busque G (2016). Subcutaneous fentanyl administration: a novel approach for pain management in a rural and suburban prehospital setting. Prehosp Emerg Care.

[134] Andolfatto G, Innes K, Dick W, Jenneson S, Willman E, Stenstrom R, et al. Prehospital analgesia with intranasal ketamine: a randomized double-blind trial in adults. Ann Emerg Med. 2019;74(2):241–50. 10.1016/j.annemergmed.2019.01.048 30926189

[135] Simons R, Strote J, Eisenberg M (2007). Emergency medical technician treatment of out-of-hospital hypoglycemia without transport...2007 Society for Academic Emergency Medicine Annual Meeting. Acad Emerg Med.

[136] Kupas DF, Miller DD (2010). Out-of-hospital chest escharotomy: a case series and procedure review. Prehosp Emerg Care.

[137] Jones C, Tzannes A, Reid C (2010). A prehospital paediatric tension viscerothorax presenting as a tension pneumothorax: a diagnostic dilemma. Emerg Med J.

[138] Battaloglu E, Porter K (2017). Management of pregnancy and obstetric complications in prehospital trauma care: faculty of prehospital care consensus guidelines. Emerg Med J.

[139] Xia S, Jones M, Perera T, Cowan E, Birnbaum A (2015). Prehospital trauma arrival notification associated with significantly more image studies in minor head trauma patients discharged from emergency department. Acad Emerg Med.

[140] Bayliss D, Oswald R (2015). Don’t fall for it. Can Paramed.

[141] Sen A, Rubinfeld I, Azuh O, Coba V, Doud A, Horst HM (2010). Point-of-care visensia (Biosign) index predicts life-saving interventions in prehospital trauma patients. Acad Emerg Med.

[142] Van Rein EAJ, Van Der Sluijs R, Voskens FJ, Lansink KWW, Houwert RM, Lichtveld RA, et al. Development and validation of a prediction model for prehospital triage of trauma patients. JAMA Surg. 2019;154(5):421–9. 10.1001/jamasurg.2018.4752 PMC6537785 30725101

[143] Lerner EB, Drendel AL, Falcone RA, Weitze KC, Badawy MK, Cooper A (2015). A consensus-based criterion standard definition for pediatric patients who needed the highest-level trauma team activation. J Trauma Acute Care Surg.

[144] Hedges J, Newgard C, Mullins R (2006). Emergency medical treatment and active labor act and trauma triage. Prehosp Emerg Care.

[145] Reavley P (2014). The challenges of pre-hospital paediatric trauma care. J Paramed Pract.

[146] Fleet R, Tounkara F, Turcotte S, Ouimet M, Dupuis G, Poitras J (2017). Rural versus urban pre-hospital and in-hospital mortality following a traumatic event in Quebec, Canada. Can J Emerg Med.

[147] Schauer SG, April MD, Hill GJ, Naylor JF, Borgman MA, De Lorenzo RA (2018). Prehospital interventions performed on pediatric trauma patients in Iraq and Afghanistan. Prehosp Emerg Care.

[148] Smith CA, Hardern RD, LeClerc S, Howes RJ (2019). Prehospital analysis of northern trauma outcome measures: the PHANTOM study. Emerg Med J.

[149] Zeller J, Fox A, Pryor JP (2008). Beyond the battlefield: the use of hemostatic dressings in civilian EMS. JEMS. J Emerg Med Serv.

[150] Schwartz RB, Reynolds BZ, Shiver SA, Lerner EB, Greenfield EM, Solis RA (2011). Comparison of two packable hemostatic gauze dressings in a porcine hemorrhage model. Prehosp Emerg Care.

[151] Boulton AJ, Lewis CT, Naumann DN, Midwinter MJ (2018). Prehospital haemostatic dressings for trauma: a systematic review. Emerg Med J.

[152] Smith AH, Laird C, Porter K, Bloch M. Haemostatic dressings in prehospital care. Emerg Med J. 2013;30(10):784–9. 10.1136/emermed-2012-201581 23161808

[153] El Sayed MJ, Tamim H, Mailhac A, Mann NC (2017). Trends and predictors of limb tourniquet use by civilian emergency medical services in the United States. Prehosp Emerg Care.

[154] Bebarta VS, Garrett N, Boudreau S, Castaneda M (2015). A prospective, randomized trial of intravenous hydroxocobalamin versus whole blood transfusion compared to no treatment for class III hemorrhagic shock resuscitation in a prehospital swine model. Acad Emerg Med.

[155] Scott N, Sutton S (2016). BET 1: give prehospital blood and save a life?. Emerg Med J.

[156] Porsi LH, Gerhardt RT (2015). Tactical study of care originating in the prehospital environment (TACSCOPE): analysis of the incidence and outcomes of traumatic pneumothorax in U.S. battlefield casualties. Acad Emerg Med.

[157] Maarouf A, McQuown CM, Frey JA, Ahmed RA, Derrick L (2017). Iatrogenic spinal cord injury in a trauma patient with ankylosing spondylitis. Prehosp Emerg Care.

[158] Warner S (2010). Spinal injury: how should we immobilize in the prehospital environment?. J Paramed Pract.

[159] Krell JM, McCoy MS, Sparto PJ, Fisher GL, Stoy WA, Hostler DP (2006). Comparison of the Ferno scoop stretcher with the long backboard for spinal immobilization. Prehosp Emerg Care.

[160] Dixon M, O'Halloran J, Cummins NM (2014). Biomechanical analysis of spinal immobilisation during prehospital extrication: a proof of concept study. Emerg Med J.

[161] Scott I, Porter K, Laird C, Greaves I, Bloch M (2014). The pre-hospital management of pelvic fractures: initial consensus statement. J Paramed Pract.

[162] O'Connor R (2019). Prehospital care in isolated neck of femur fracture: a literature review. J Paramed Pract.

[163] von Vopelius-Feldt J, Wood J, Benger J (2014). Critical care paramedics: where is the evidence? A systematic review. Emerg Med J.

[164] Cummins NM, Dixon M, Garavan C, Landymore E, Mulligan N, O'Donnell C (2013). Can advanced paramedics in the field diagnose patients and predict hospital admission?. Emerg Med J.

[165] Wilson C, Harley C, Steels S (2018). Systematic review and meta-analysis of pre-hospital diagnostic accuracy studies. Emerg Med J.

[166] Andrew E, Nehme Z, Bernard S, Smith K (2018). Pediatric anaphylaxis in the prehospital setting: incidence, characteristics, and management. Prehosp Emerg Care.

[167] Tiyyagura GK, Arnold L, Cone DC, Langhan M (2014). Pediatric anaphylaxis management in the prehospital setting. Prehosp Emerg Care.

[168] Chung T, Lovstrom L, Vandenberghe C, Couperthwaite S, Sookram S, Liss K (2013). Prehospital anaphylaxis mimics in one Canadian urban centre. Can J Emerg Med.

[169] Lammers RL, Byrwa MJ, Fales WD, Hale RA (2009). Simulation-based assessment of paramedic pediatric resuscitation skills. Prehosp Emerg Care.

[170] Travers A, Green R, Cain E, Campbell SG, Jensen JL, Petrie D (2013). Can paramedics diagnose sepsis in the prehospital setting? A feasibility study. Can J Emerg Med.

[171] Ericsson A, Frenckner B, Broman LM (2017). Adverse events during inter-hospital transports on extracorporeal membrane oxygenation. Prehosp Emerg Care.

[172] Peate I (2016). Primum non nocere: first, do no harm. J Paramed Pract.

[173] Mendes A (2018). Clinical skills in paramedic practice. J Paramed Pract.

[174] BET 2 (2011). Which intraosseous device is best in the prehospital setting?. Emerg Med J.

[175] Bullard-Berent J (2009). Intraosseous infusion: an instructional program for healthcare providers, revised edition (CD-ROM). Prehosp Emerg Care.

[176] Salzman JG, Loken NM, Wewerka SS, Burnett AM, Zagar AE, Griffith KR (2017). Intraosseous pressure monitoring in healthy volunteers. Prehosp Emerg Care.

[177] Frisch A, Cammarata S, Mosesso VN, Martin-Gill C (2013). Multivariate analysis of successful intravenous line placement in the prehospital setting. Prehosp Emerg Care.

[178] Garwe T, Johnson JJ, Letton RW (2016). Indication bias explains some of the observed increased mortality associated with use of prehospital intravenous fluids in a pediatric trauma population. Acad Emerg Med.

[179] Karl A, Pham T, Yanosky JD, Lubin J (2016). Variability of Uncrossmatched blood use by helicopter EMS programs in the United States. Prehosp Emerg Care.

[180] Yates AM, Dunn CS, Hostler D (2007). Evaluation of respiratory function during reeves stretcher use. Prehosp Emerg Care.

[181] Hale KE, Gavin C, O'Driscoll BR (2008). Audit of oxygen use in emergency ambulances and in a hospital emergency department. Emerg Med J.

[182] Mattera C. The physiology of respirations with CPAP. JEMS. 2011 Supp:6–10.

[183] Baruch EN, Kragh JF, Berg AL, Aden JK, Benov A, Shina A (2017). Confidence–competence mismatch and reasons for failure of non-medical tourniquet users. Prehosp Emerg Care.

[184] Galinski M, Petrovic T, Rodrigues A, Hermann M, Catineau J, Adnet F (2010). Out-of-hospital diagnosis of a ruptured ectopic pregnancy: myometrial embryo implantation, an exceptional diagnosis. Prehosp Emerg Care.

[185] Jensen JL, Tavares W, Calder LA, Bienkowski A, Walker M, Travers A (2014). Experiential and rational decision-making: a survey to determine decision-making styles of paramedics and paramedic students. Can J Emerg Med.

[186] Jensen JL, Bienkowski A, Travers AH, Calder LA, Walker M, Tavares W (2016). A survey to determine decision-making styles of working paramedics and student paramedics. Can J Emerg Med.

[187] Reay G, Rankin JA, Smith-MacDonald L, Lazarenko GC (2018). Creative adapting in a fluid environment: an explanatory model of paramedic decision making in the pre-hospital setting. BMC Emerg Med.

[188] Carlson JN, Karns C, Mann NC, Jacobson KE, Dai M, Colleran C (2016). Procedures performed by emergency medical Services in the United States. Prehosp Emerg Care.

[189] Ingalsbe G, Cienki J, Schrank K (2012). Emergency medical service providers perspectives on management of the morbidly obese. Acad Emerg Med.

[190] Noveanu J, Amsler F, Ummenhofer W, von Wyl T, Zuercher M (2017). Assessment of simulated emergency scenarios: are trained observers necessary?. Prehosp Emerg Care.

[191] Mancera M, Gussick M, Lohmeier M, Shah MN, Thompson R (2018). Acceptability of simulation-based education among emergency medical services providers. Acad Emerg Med.

[192] McCarthy J, Spain A, Varner L (2018). Supporting safety 3: simulations supporting ambulance safety & mental health. JEMS.

[193] Studnek JR, Fernandez AR, Margolis GS (2009). Assessing continued cognitive competence among rural emergency medical technicians. Prehosp Emerg Care.

[194] Smart G (2011). I.F.E.A.R reflection: an easy to use, adaptable template for paramedics. J Paramed Pract.

[195] Kollek D, Wanger K, Welsford M (2009). Chemical, biological, radiological and nuclear preparedness training for emergency medical services provider. CJEM..

[196] Chaput CJ, Deluhery MR, Stake CE, Martens KA, Cichon ME (2007). Disaster training for prehospital providers. Prehosp Emerg Care.

[197] Olasveengen TM, Tomlinson AE, Wik L, Sunde K, Steen PA, Myklebust H (2007). A failed attempt to improve quality of out-of-hospital CPR through performance evaluation. Prehosp Emerg Care.

[198] Morrison L, Cassidy LF, Welsford M, Chan TM (2016). Clinical performance feedback to paramedics: what they receive and what they need. Can J Emerg Med.

[199] Benoit J, Widmeier K, McMullan J (2017). Evaluate clinical research with these seven questions. JEMS.

[200] Griffin D (2015). Paramedic prescribing: a potion for success or a bitter pill to swallow?. J Paramed Pract.

[201] Hleboff G (2018). Stress inoculation training: paramedic instructor buzz-word or meaningful tool?. Can Paramed.

[202] Bentley MA, Eggerichs-Purcell JJ, Brown William E, Wagoner R, Gibson GC, Sahni R (2013). A national assessment of the roles and responsibilities of training officers. Prehosp Emerg Care.

[203] D'Alessandro C (2018). Take a seat, let's talk about pocus. Can Paramed.

[204] McQueen C, Wyse M (2014). The delivery of the new prehospital emergency medicine curriculum: reflections on a pilot programme in the UK. Emerg Med J.

[205] Maloney LM, Dilger JP, Werfel PA, Cimino LM (2017). Facilitating higher order learning in emergency medical technician students using teaching strategies that reinforce adult learning principles. Acad Emerg Med.

[206] Rutherford G, Inglis D (2010). Outlining the diploma in immediate medical care. J Paramed Pract.

[207] Matheson R (2019). Human factors in student paramedic practice. J Paramed Pract.

[208] Theriault R (2017). Virtual reality in paramedic education. Can Paramed.

[209] Chan J, Griffith LE, Costa AP, Leyenaar MS, Agarwal G. Community paramedicine: a systematic review of program descriptions and training. CJEM. 2019;21(6):749–61. 10.1017/cem.2019.14 30885280

[210] Hughes G (2011). Critiquing critical care paramedics. Emerg Med J.

[211] Myers JA, Haney MF, Griffiths RF, Pierse NF, Powell DMC (2015). Fatigue in air medical clinicians undertaking high-acuity patient transports. Prehosp Emerg Care.

[212] James FO, Waggoner LB, Weiss PM, Patterson PD, Higgins JS, Lang ES (2018). Does implementation of biomathematical models mitigate fatigue and fatigue-related risks in emergency medical services operations? A systematic review. Prehosp Emerg Care.

[213] Temple JL, Hostler D, Martin-Gill C, Moore Charity G, Weiss PM, Sequeira DJ (2018). Systematic review and meta-analysis of the effects of caffeine in fatigued shift workers: implications for emergency medical services personnel. Prehosp Emerg Care.

[214] Patterson PD, Weaver MD, Fabio A, Teasley EM, Renn ML, Curtis BR (2018). Reliability and validity of survey instruments to measure work-related fatigue in the emergency medical services setting: a systematic review. Prehosp Emerg Care.

[215] Barger LK, Runyon MS, Renn ML, Moore CG, Weiss PM, Condle JP (2018). Effect of fatigue training on safety, fatigue, and sleep in emergency medical services personnel and other shift workers: a systematic review and meta-analysis. Prehosp Emerg Care.

[216] Patterson PD, Buysse DJ, Weaver MD, Callaway CW, Yealy DM (2015). Recovery between work shifts among emergency medical services clinicians. Prehosp Emerg Care.

[217] Martin-Gill C, Barger LK, Moore CG, Higgins JS, Teasley EM, Weiss PM (2018). Effects of napping during shift work on sleepiness and performance in emergency medical services personnel and similar shift workers: a systematic review and meta-analysis. Prehosp Emerg Care.

[218] Patterson PD, Runyon MS, Higgins JS, Weaver MD, Teasley EM, Kroemer AJ (2018). Shorter versus longer shift durations to mitigate fatigue and fatigue-related risks in emergency medical services personnel and related shift workers: a systematic review. Prehosp Emerg Care.

[219] Studnek JR, Infinger AE, Renn ML, Weiss PM, Condle JP, Flickinger KL (2018). Effect of task load interventions on fatigue in emergency medical services personnel and other shift workers: a systematic review. Prehosp Emerg Care.

[220] Patterson PD, Weaver MD, Frank RC, Warner CW, Martin-Gill C, Guyette FX (2012). Association between poor sleep, fatigue, and safety outcomes in emergency medical services providers. Prehosp Emerg Care.

[221] Thompson S, Armstrong K, Kirk A (2017). Providing support to students following a mass casualty incident. J Paramed Pract.

[222] Gouweloos-Trines J, Tyler MP, Giummarra MJ, Kassam-Adams N, Landolt MA, Kleber RJ (2017). Perceived support at work after critical incidents and its relation to psychological distress: a survey among prehospital providers. Emerg Med J.

[223] Halpern J, Maunder RG, Schwartz B, Gurevich M (2012). The critical incident inventory: characteristics of incidents which affect emergency medical technicians and paramedics. BMC Emerg Med.

[224] Deakins A, Jones-Keyte L, Brown P (2019). Honouring inclusivity and support in paramedicine. J Paramed Pract.

[225] Johnston S (2018). My time as a paramedic and why mental health matters. J Paramed Pract.

[226] Billingham M (2017). Rural and remote paramedicine -- our dirty little secret. Can Paramed.

[227] Heightman AJ (2007). Honoring our own: the national EMS memorial service. JEMS.

[228] Gallagher A, Zasada M, Jago R, Austin Z, Banks S, Lucas G (2018). Fitness-to-practise concerns and preventative strategies. J Paramed Pract.

[229] Sparre M (2016). How can paramedics stay fit and healthy whilst working?. Can Paramed.

[230] Jones A, Hignett S (2007). Safe access/egress systems for emergency ambulances. Emerg Med J.

[231] Fass B. Reducing lift injuries. JEMS. 2017;42(10):47–51.

[232] Lynch MJ, Suyama J, Guyette FX (2018). Scene safety and force protection in the era of ultra-potent opioids. Prehosp Emerg Care.

[233] Smith DL, Petruzzello SJ, Goldstein E, Ahmad U, Tangella K, Freund GG (2011). Effect of live-fire training drills on firefighters? Platelet number and function. Prehosp Emerg Care.

[234] Creed JO, Cyr JM, Owino H, Box SE, Ives-Rublee M, Sheitman BB (2018). Acute crisis care for patients with mental health crises: initial assessment of an innovative prehospital alternative destination program in North Carolina. Prehosp Emerg Care.

[235] Yau L, Mukarram MA, Kim S, Arcot K, Thavorn K, Taljaard M (2016). Outcomes and resource utilization among syncope patients transported by emergency medical services. Can J Emerg Med.

[236] Miles J, O'Keeffe C, Jacques R, Stone T, Mason S (2017). 17 Exploring ambulance conveyances to the emergency department: a descriptive analysis of non-urgent transports. Emerg Med J.

[237] Stewart KE, Cowan LD, Thompson DM, Sacra JC (2011). Factors at the scene of injury associated with air versus ground transport to definitive care in a state with a large rural population. Prehosp Emerg Care.

[238] Wandling MW, Haut ER (2018). Dangers of private vehicle transportation vs emergency medical services transportation-reply. JAMA Surg.

[239] Jones R, Langford S (2015). Australia’s flying doctors. How the world’s largest aeromedical response service provides effective patient retrieval in the outback. JEMS.

[240] American College of Emergency P, National Association of EMSP, Air Medical Physician A, Association of Air Medical S, National Association of State EMSO (2008). Air ambulance medical transport advertising and marketing. Ann Emerg Med.

[241] Genes N, Hwang U, Handel DA, Pines J, Aronsky D, Ginde AA (2011). Predictors of ambulance diversion in nine emergency departments. Acad Emerg Med.

[242] Handel DA, Pines J, Aronsky D, Genes N, Ginde AA, Hackman J (2011). Variations in crowding and ambulance diversion in nine emergency departments. Acad Emerg Med.

[243] Sun J, Silvestri S, Papa L, Diaz L, Swinghome M, Ralls G (2007). The impact of emergency department paramedic staffing on emergency medical services unit off-load time...2007 Society for Academic Emergency Medicine Annual Meeting. Acad Emerg Med.

[244] Patel PB, Vinson DR (2011). Ambulance diversion reduction and elimination: the 3-2-1 plan. Acad Emerg Med.

[245] Steer S, Bhalla MC, Zalewski J, Frey J, Nguyen V, Mencl F (2016). Use of radio frequency identification to establish emergency medical service offload times. Prehosp Emerg Care.

[246] Stewart K, Garwe T, Bhandari N, Danford B, Albrecht R (2016). Factors associated with the use of helicopter inter-facility transport of trauma patients to tertiary trauma centers within an organized rural trauma system. Prehosp Emerg Care.

[247] Krebs W, Higgins T, Buckley M, Augustine JJ, Raetzke BD, Werman HA (2019). Botulism outbreak in a regional community hospital: lessons learned in transfer and transport considerations. Prehosp Emerg Care.

[248] Rouse J (2016). What do ambulance service personnel perceive to be the process of and issues with inter-hospital transfers?. J Paramed Pract.

[249] Davis M, Lewell M, McLeod S, Dukelow A (2014). A prospective evaluation of the utility of the prehospital 12-lead electrocardiogram to change patient management in the emergency department. Prehosp Emerg Care.

[250] Nam J, Caners K, Bowen JM, Welsford M, O'Reilly D (2014). Systematic review and meta-analysis of the benefits of out-of-hospital 12-lead ECG and advance notification in ST-segment elevation myocardial infarction patients. Ann Emerg Med.

[251] Fitzpatrick D, McLean S (2010). Reperfusion of old or new: left bundle branch block?. J Paramed Pract.

[252] Hanson TC, Williamson D. Identifying barriers to prehospital thrombolysis in the treatment of acute myocardial infarction. Emerg Med J. 2006 ;23(8):650–3. 10.1136/emj.2005.033993 PMC2564177 16858108

[253] Trojanowski J, Macdonald RD (2011). Safe transport of patients with acute coronary syndrome or cardiogenic shock by skilled air medical crews. Prehosp Emerg Care.

[254] Mitchell C, Perkins Z (2007). Prehospital thrombolysis of acute myocardial infarction in dextrocardia. Emerg Med J.

[255] Robichaud L, Ross D, Proulx MH, Legare S, Vacon C, Xue X (2016). Prehospital nitroglycerin safety in inferior ST elevation myocardial infarction. Prehosp Emerg Care.

[256] Burman RA, Zakariassen E, Hunskaar S (2011). Acute chest pain - a prospective population based study of contacts to Norwegian emergency medical communication centres. BMC Emerg Med.

[257] Stenestrand U, Lindback J, Wallentin L (2006). Long-term outcome of primary percutaneous coronary intervention vs prehospital and in-hospital thrombolysis for patients with ST-elevation myocardial infarction. JAMA.

[258] Kirtane AJ, Harrington RA (2017). One-year follow-up of the european ambulance acute coronary syndrome angiography trial. JAMA Cardiol.

[259] Swor R, Hegerberg S, McHugh-Mcnally A, Goldstein M, McEachin C (2006). Prehospital 12-lead ECG: efficacy or effectiveness?. Prehosp Emerg Care.

[260] Cone DC, Lee CH, Van Gelder C (2013). EMS activation of the cardiac catheterization laboratory is associated with process improvements in the care of myocardial infarction patients. Prehosp Emerg Care.

[261] Segal E, Ross D, Proulx M, Xue X, Vacon C (2015). ‘False leads’: derivation and validation of a rule to minimize falsepositive prehospital cath lab activations for STEMI. Can J Emerg Med.

[262] Turnipseed SD, Amsterdam EA, Laurin EG, Lichty LL, Miles PH, Diercks DB (2010). Frequency of non-ST-segment elevation injury patterns on prehospital electrocardiograms. Prehosp Emerg Care.

[263] Russi CS, Myers LA, Kolb LJ, Steever K, Nestler DM, Bjerke MC (2011). Prehospital diagnosis of st-segment elevation myocardial infarction using an “all-posterior” 12-lead electrocardiogram. Prehosp Emerg Care.

[264] Ross DW, Cooperrider C, Homan MB (2014). Acute coronary ischemia identified by EMS providers in a standing middle-aged male with atypical symptoms. Prehosp Emerg Care.

[265] Youngquist ST, McIntosh SE, Swanson ER, Barton ED (2010). Air ambulance transport times and advanced cardiac life support interventions during the interfacility transfer of patients with acute ST-segment elevation myocardial infarction. Prehosp Emerg Care.

[266] Shen YC, Hsia RY (2011). Ambulance diversion and survival among patients with acute myocardial infarction - reply. JAMA..

[267] Smith KA, High K, Collins SP, Self WH (2015). A preprocedural checklist improves the safety of emergency department intubation of trauma patients. Acad Emerg Med.

[268] Stephens CT, Kahntroff S, Dutton RP (2009). The success of emergency endotracheal intubation in trauma patients: a 10-year experience at a major adult trauma referral center. Anesth Analg.

[269] Chapman CA, Bicca-Marques JC, Calvignac-Spencer S, Fan P, Fashing PJ, Gogarten J (1916). Games academics play and their consequences: how authorship, h-index and journal impact factors are shaping the future of academia. Proc R Soc B.

[270] Makrides T, Ross L, Gosling C, Acker J, O’Meara P. From stretcher bearer to practitioner: a brief narrative review of the history of the Anglo-American paramedic system. Austral Emerg Care. 2022;25(4):347–53. 10.1016/j.auec.2022.05.001 35659867

[271] Makrides T, Ross L, Gosling C, Acker J, O’Meara P. Defining two novel sub models of the Anglo-American paramedic system: a Delphi study. Austral Emerg Care. 2021;25(3):229–34. 10.1016/j.auec.2021.11.001 34838505

